# Human induced pluripotent stem cells as a tool for disease modeling and drug screening for COVID-19

**DOI:** 10.1590/1678-4685-GMB-2020-0198

**Published:** 2020-11-16

**Authors:** Patricia Nolasco, Juliana Borsoi, Carolina Borsoi Moraes, Lucio H. Freitas-Junior, Lygia Veiga Pereira

**Affiliations:** 1 Universidade de São Paulo, Instituto de Biociências, Departamento de Genética e Biologia Evolutiva, Laboratório Nacional de Células-tronco Embrionárias (LaNCE), São Paulo, SP, Brazil.; 2 Universidade Federal de São Paulo, Departamento de Ciências Farmacêuticas, Diadema, SP, Brazil.; 3 Universidade de São Paulo, Instituto de Ciências Biomédicas, Departamento de Microbiologia, Phenotypic Screening Platform, São Paulo, SP, Brazil.

**Keywords:** Stem cell differentiation, COVID-19, drug screening, Brazil, human pluripotent stem cells

## Abstract

The emergence of the new corona virus (SARS-CoV-2) and the resulting COVID-19 pandemic requires fast development of novel prevention and therapeutic strategies. These rely on understanding the biology of the virus and its interaction with the host, and on agnostic phenotypic screening for compounds that prevent viral infection. *In vitro* screenings of compounds are usually performed in human or animal-derived tumor or immortalized cell lines due to their ease of culturing. However, these platforms may not represent the tissues affected by the disease *in vivo*, and therefore better models are needed to validate and expedite drug development, especially in face of the COVID-19 pandemic. In this scenario, human induced pluripotent stem cells (hiPSCs) are a powerful research tool due to their ability to generate normal differentiated cell types relevant for the disease. Here we discuss the different ways hiPSCs can contribute to COVID-19 related research, including modeling the disease *in vitro* and serving as a platform for drug screening.

## Introduction

The COVID-19 (Coronavirus Disease 2019) pandemic, caused by SARS-CoV-2, is a global emergency that affects countries worldwide and continues to spread rapidly ( Livingston *et al.* 2020; World Health Organization 2020). On July 24th, 2020, Brazil had the second-highest number of COVID-19 cases and number of deaths, behind only the USA (Dong *et al.* 2020). Being so severely affected, it is essential for the country to outline an action plan aiming to understand the biology of the virus and its interaction with the host which will eventually lead to the development of novel vaccines and therapies against the disease. In this review we discuss the role of human induced pluripotent stem cells (hiPSCs) for basic research and screening for drugs against SARS-CoV-2. 

## Drug Screening and COVID-19

Antiviral chemotherapy is a much sought-after strategy for control of COVID-19. However, this is a new disease for which there is scarce knowledge, and *in vitro* and *in vivo* models are not readily available. In the absence of an effective vaccine, drug discovery for the development of novel and COVID-19-specific antivirals is ideal. However, *de novo* drug discovery is a lengthy process, and thus, a strategy that is being pursued in parallel and may expedite the development of COVID-19 chemotherapy is drug repositioning or repurposing. This approach has been used with success in the past for several diseases, including viral infections. In fact, the first treatment approved by the FDA for HIV, Zidovudine (AZT, azidothymidine), was originally used in cancer treatment (Richman 1988; Maeda *et al.* 2019).

Usually drug discovery begins with screening, and modern programs in both Academia and Industry rely on two different approaches for interrogating compound activity, often used in complement to each other: target-based and cell-based or phenotypic screening (Moffat *et al.* 2017). The former refers to screening assays that normally measure compound activity (inhibition, activation) or binding in a biochemical assay with a single isolated molecular target, ideally previously shown to be important in the resolution of the disease and validated genetically or by chemical methods. The latter refers to assays that use cells, tissues, or even whole organisms, to measure compound capacity to modulate a phenotype that correlates with efficacious chemotherapy of the disease of interest, without previous knowledge of the target. While both strategies have their own advantages and limitations, phenotypic screening has been shown to outperform target-based screening in the discovery of novel “first in class” drugs, as they allow for the discovery of molecules on unknown targets, with novel molecular mechanisms of action - sometimes even with a multitarget or pleiotropic mechanism of action (Hopkins 2008). Phenotypic screening assays are also considered more physiologically relevant, as molecules are interrogated in a cellular milieu, and the phenotype measured in assay endpoint is usually closer to the efficacy endpoint measured later in the clinic (Swinney and Anthony 2011). One disadvantage of phenotypic screenings is that the molecular target of active compounds is often unknown and requires further work for their discovery - a process usually referred to as target deconvolution (Schirle and Jenkins 2016). In the case of COVID-19, an emerging disease, phenotypic screening can offer the possibility of discovering new drugs and repurposing existing ones, targeting either SARS-CoV-2 or host proteins and pathways. Viral proteins, as well as cellular receptors and pathways used by the virus might be amenable to modulation/inhibition by existing chemotherapies, thus expediting the development of new drugs. In fact, several FDA-approved drugs have already been proposed for repositioning for COVID-19 based on phenotypic screening campaigns carried out since the beginning of the pandemic (Riva *et al.* 2020; Heiser *et al.* 2020; Jeon *et al.* 2020; Mirabelli *et al.* 2020). However, these candidates still require demonstration of clinical efficacy to be repurposed for COVID-19 chemotherapy and currently there are several clinical trials evaluating candidates for COVID-19 chemotherapy. 

Another aspect is the physiological relevance of the screening assay and the cell-based model adopted, as the cellular assay should try to replicate key aspects of the disease. *In vitro* screenings of compounds are usually performed in models that are easier to manipulate, such as tumor or immortalized cell lines like HeLa (Human cervix epitheloid carcinoma), CHO (Chinese Hamster Ovary cells) and HCT116 (human colorectal carcinoma) (Zhang *et al.* 2012). The choice of a cell line for phenotypic screening impacts on the results and drugs found to have the desired activity profile, the hits*.* So much so that compounds can have a remarkable difference in activity depending on the cell model used: a recent study showed that chloroquine may or may not inhibit SARS-CoV-2 infection *in vitro* depending on the cell model used (Hoffmann *et al.* 2020b).

However, even when working with relevant-tissue type cells, these lineages often do not represent or mimic the tissues affected by the disease *in vivo*. Thus, for the discovery/repositioning process of compounds to be more efficient, *in vitro* models that translate, in the most reliable way possible, the conditions observed *in vivo* are extremely important. 

## Human pluripotent stem cells as a study model for COVID-19

Pluripotent stem cells (PSC) are defined by their capability of differentiating into cell types derived from the three embryonic germ layers. Initially established in culture as embryonic stem cells (ESCs) derived from the inner cell mass of mouse blastocysts (Evans and Kaufman 1981; Martin 1981), these cells can be maintained practically indefinitely in culture in an undifferentiated state. Upon induction of differentiation *in vitro*, they can specialize in potentially any tissue of the adult (Shi *et al.* 2017). Although embryo-derived human ESCs (Thomson *et al.* 1998) are equivalently pluripotent, the development of methods to reprogram adult somatic cells to pluripotency has revolutionized the stem cell field (Takahashi and Yamanaka 2006; Takahashi *et al.* 2007). Human induced pluripotent stem cells (hiPSCs) can be derived from any individual with a phenotype/genotype of interest, providing a potent tool for *in vitro* disease modeling. Patient-specific hiPSC models make it possible to obtain differentiated cell types relevant for a particular disease, such as neurons or cardiomyocytes, that can be used to understand molecular mechanisms of pathogenesis and as a platform for drug screening.

More recently, hiPSC-derived cells have been shown to be valuable also as a model for infectious diseases (Shi *et al.* 2017). Our group has shown the infection capacity of the protozoan *Trypanosoma cruzi* in hiPSC-derived cardiomyocytes (iCMs), demonstrating the potential of these cells as a human model for studying cardiomyopathy in Chagas disease and also for the development of new therapies against the parasite (da Silva Lara *et al.* 2018). Similarly, hiPSC-derived hepatocytes infected with hepatitis B virus (Xia *et al.* 2017), hPSC-Neural progenitors infected with Zika virus (Zhou *et al.* 2017) and Herpes Simplex Virus-1 (D’Aiuto *et al.* 2017) have been reported. Also, infection susceptibility of numerous hiPSC-derived cell types with SARS-CoV-2 have already been demonstrated (Monteil *et al.* 2020; Yang L *et al.* 2020; Sharma *et al.* 2020). Table 1 shows the advantages and disadvantages of this model in comparison to others used during the drug development process.

**Table 1 -:** Advantages and disadvantages of the different models used for drug development.

Model	Advantages	Disadvantages
hiPSC-derived cells	Human model, patient (genotype)-specific, suitable for gene editing	Labor/time consuming and higher cost culture
	Can generate multiple relevant cell-types with same genetic background	Some differentiated cell types with fetal phenotype
	Increased complexity can be achieved with co-culture and organoids	Possible variations among batches of differentiations
Human cancer/ immortalized lines	Easy to keep in culture and maintain	Fail to represent or mimic the tissues affected by the disease *in vivo*
	Passage number is not a concern	Carry tumor-associated mutations, such as P53 mutations; some cell lines have mutations in genes controlling the innate immune response
Transgenic mice	Complete organism able to show systemic disease	Distinct from human biology and unlikely to capture key aspects of some diseases
	Can be engineered to express molecules of interest	Use increasingly discouraged for ethical reasons

## Relevant cell types in the COVID-19 study

SARS-CoV-2 infection is mainly caused by the binding of the receptor-binding domain (RBD) of the spike (S) viral surface protein to the human angiotensin I converting enzyme 2 (ACE2) receptor, an interaction that occurs with high affinity (Hoffmann *et al.* 2020a; Shang *et al.* 2020a; Shang *et al.* 2020b; Wan *et al.* 2020; Zhang H *et al.* 2020; Walls *et al.* 2020). The host cell TMPRSS2 transmembrane protease action is also crucial, since it cleaves residues of the viral spike protein and allows the fusion of viral and cellular membranes (Matsuyama *et al.* 2020; Hoffmann *et al.* 2020a). Low expression of this protein does not protect against infection, since a furin preactivation can also facilitate viral entry in some types of cells regardless of TMPRSS2 expression levels (Shang *et al.* 2020a). Additionally, in HEK293 cells stably expressing ACE2, it was demonstrated that viral entry happens mainly through endocytosis, and that other proteases, such as cathepsin L, are critical (Ou *et al.* 2020). These data suggest that the range of possible infection mechanisms are broader than initially thought and can vary among different cell types.

ACE2 is expressed in the lungs (mainly in type 2 alveolar epithelial cells - AT2s) and also in the heart, intestine and kidneys, supporting mechanisms for dysfunction in multiple organs, which has also been observed in COVID-19 patients (Zhang H *et al.* 2020). The TMPRSS2 protease has high expression in the respiratory tract epithelium, a fact that justifies the tropism of SARS-CoV-2 to lungs; in addition, it is expressed in prostate, colon, stomach and salivary gland (Vaarala *et al.* 2001; Matsuyama *et al.* 2020; Hoffmann *et al.* 2020a). Despite representing the majority of infected cells by SARS-CoV-2 (Zheng *et al.* 2020), single cell RNA sequencing of pulmonary cells has shown that the expression of *ACE2* is restricted to only a small subset (around 1.4%) of AT2s (Ziegler *et al.* 2020; Ackermann *et al.* 2020; Zhao *et al.* 2020), while expression of *TMPRSS2* is found in approximately 30% of the same cell type (Ziegler *et al.* 2020; Lukassen *et al.* 2020). Co-expression of both *ACE2* and *TMPRSS2* was found mainly in AT2 and ciliated cells among all the cell types analyzed from fibrotic lung tissue, but at very low rates of 0.8% and 5.3%, respectively (Ziegler *et al.* 2020). These data show that, although the functional role of both genes in COVID-19 seems established, there is still much to learn about the influence of expression rates in susceptibility of infection and also in the complexity of disease outcome. For that, together with clinical data, both *in vitro* and *in vivo* models will be crucial.

In the lungs, AT2s perform central functions, the main one being the production and secretion of surfactants that prevent the collapse of the pulmonary tissue by regulating the surface tension of the alveoli (reviewed by Fehrenbach, 2001). They also proliferate and can differentiate into type 1 alveolar epithelial cells (AT1s) after lung injury and play a role in immune defense upon infection (Fehrenbach 2001; Jacob *et al.* 2019). Despite the fact that AT2s are highly proliferative in a renewing tissue, primary AT2s proliferate poorly *in vitro* and last only a few passages in the absence of mesenchymal support. In contrast, hiPSC-derived AT2s (iAT2s) can form alveolar organoids with proliferative potential, morphology, and molecular phenotype comparable to lung alveoli (Jacob *et al.* 2017; Jacob *et al.* 2019). Correction of a mutation in the surfactant protein-B encoding gene (*SFTPB*) in a patient-specific cell line restored the surfactant production and processing of the respective iAT2s, demonstrating the suitability of these cells for pulmonary disease modeling (Jacob *et al.* 2017). Thus, generation of AT2s from hiPSCs provides a powerful tool to model COVID-19 pulmonary phenotypes *in vitro* and to investigate the cellular and molecular changes in these cells after infection by SARS-CoV-2, as showed recently in a model of at air-liquid interface witch mimetic the lung environment (Abo *et al.* 2020). The screening of compounds against the virus in iAT2s 3D cultures is also a possibility, although phenotypic evaluation of 3D organoids is harder to manage in high content screening platforms in comparison to monolayer cell cultures (Li *et al.* 2016). Therefore, this highly relevant physiological model is more likely to be introduced as low throughput, downstream, secondary assays for validating and profiling antiviral activity and mechanism of action of compounds.

Infection of the endothelium by SARS-CoV-2 has also been proposed as a mechanism of viral migration to different organs (Monteil *et al.* 2020). Post-mortem histology revealed lymphocytic endotheliitis in lung (Ackermann *et al.* 2020), heart (Tavazzi *et al.* 2020), kidney, liver and in the submucosal vessels of the small intestine (Varga *et al.* 2020) of COVID-19 patients. Specifically, in endothelial cells, direct infection was reported by one study (Ackermann *et al.* 2020), despite the fact that this cell type usually has low levels of *ACE2* expression (Nicin *et al.* 2020; Ackermann *et al.* 2020). Recently, Yang L *et al*. (2020) demonstrated that, although ACE2 was detected in endothelial cells differentiated from hiPSCs (iECs), infection by SARS-CoV-2 pseudo-entry virus was very low in comparison to other cells types, such as cardiomyocytes and dopaminergic neurons. In a pilot experiment, our group also observed no infection of iECs by SARS-CoV-2, even though (low) *ACE2* expression was detected (unpublished data). These data strengthen the hypothesis that other factors, apart from expression of this receptor, may play a role for viral entry to occur. Furthermore, it is important to consider that, *in vivo*, the systemic response against infection could trigger similar molecular changes in endothelial cells as observed for nasal/pulmonary epithelial cells, for which it was shown that *ACE2* is an interferon stimulated gene (ISG) (Ziegler *et al.* 2020). Interferons (IFNs) and ISGs are normally expressed in host cells as an antiviral and tissue-protective mechanism (Acharya *et al.* 2020; Ziegler *et al.* 2020), and it is hypothesized that upregulation of *ACE2* by these responses could favor further SARS-CoV-2 infection in severe COVID-19 cases. However, although expression of ISG was shown to be increased in the respiratory tract of COVID-19 patients (Zhou *et al.* 2020) and IFN signaling was shown to be upregulated in ﻿primary ﻿human lung epithelial cells infected with SARS-CoV-2 (Mulay *et al.* 2020), the kinetics of IFN activation in the disease remains controversial (Acharya *et al.* 2020), as the same responses were not observed in infected primary cultures of dendritic cells and macrophages, for example (Yang D *et al.* 2020). In particular, the need of previous activation of endothelial cells by IFNs and/or proinflammatory cytokines to support direct SARS-CoV-2 infection remains uncertain and could also be addressed using iECs.

Another relevant fact is that, increasingly, cases of pulmonary embolisms and also microthrombosis in other organs have been reported in COVID-19 patients (Zuckier *et al.* 2020; Danzi *et al.* 2020; Dolhnikoff *et al.* 2020; Griffin *et al.* 2020). In line with that, anticoagulant treatment, mainly with heparin, has been shown as potentially beneficial in a subset of severe cases (Thachil 2020; Negri *et al.* 2020; Tang *et al.* 2020). Although a hypercoagulable state due to cytokine storm and indirect endothelial dysfunction is commonly observed in patients with infections, the same severe scenario was not observed in SARS patients (Madjid *et al.* 2020; Dolhnikoff *et al.* 2020) or in cases of acute respiratory distress syndrome (ARDS) secondary to influenza A (H1N1), where the presence of alveolar capillary microthrombi was 9 times less prevalent than in COVID-19 patients (Ackermann *et al.* 2020). The possible role of microcirculation abnormalities in hypoxia lead to the latter proposal of COVID-19 as a vascular-thrombotic disease instead of solely a respiratory disease. If confirmed, this would represent a paradigm shift that could possibly change the way new therapies are sought and developed. For that, iECs would also be very useful as an *in vitro* model system.

In addition to vascular dysfunction, the occurrence of cardiac manifestations in patients affected by COVID-19 (Zheng *et al.* 2020; Clerkin *et al.* 2020) also raises the hypothesis of direct cardiac myocyte infection by SARS-CoV-2 (Gallagher *et al.* 2008; Gomes *et al.* 2012). *ACE2* is highly expressed in adult human hearts, especially by cardiomyocytes (Nicin *et al.* 2020), which indicates an intrinsic susceptibility of this organ to SARS-CoV-2. It has been shown that patients with heart failure disease exhibited increased expression of this gene and might have a higher possibility of developing a heart attack and progressing to severe condition upon SARS-CoV-2 infection (Chen *et al.* 2020). Also, high doses of highly sensitive troponin-I (hs-cTnI) have been found to predict a worse COVID-19 outcome (Madjid *et al.* 2020; Ruan *et al.* 2020; Wang D *et al.* 2020; Huang *et al.* 2020; Zhou *et al.* 2020), and in a group of patients treated in China, around 10% of those who died suffered heart damage, even when no previous cardiovascular disease was present (reviewed by Zheng *et al.*, 2020). Myocardial injury, arrhythmias and viral myocarditis were also highly prevalent in groups of patients admitted to intensive care units (ICU) (Madjid *et al.* 2020; Zheng *et al.* 2020). In a case report, Tavazzi *et al*. (2020) show evidence of myocardial localization of viral particles in one patient with cardiogenic shock, although no direct cardiomyocyte or endothelial infection was observed. Therefore, it is not known whether all these cardiac alterations described are directly caused by viral infection of heart cells or were a secondary effect of impaired pulmonary function and/or systemic inflammatory state. Another question that remains open is whether patients with comorbidities have naturally increased *ACE2* expression and/or whether the use of ACE inhibitors or angiotensin II receptor blockers (ARBs) would lead to this hypothetical increase (Fang *et al.* 2020; Zheng *et al.* 2020; Clerkin *et al.* 2020; Pinto *et al.* 2020).

Recently, our group (unpublished data) and others (Yang L *et al.* 2020; Sharma *et al.* 2020) have shown that hiPSC-derived cardiomyocytes (iCMs) are susceptible to SARS-CoV-2 infection. Also, Sharma and colleagues observed that infection caused apoptosis and contractility alterations, validating these cells as a potential model to study SARS-CoV-2-related myocarditis and for phenotypic drug screening.

## Host genetics

The host response to a virus is generally not uniform, and infections can inflict different degrees of morbidity and mortality in different patients. For COVID-19, up to now, it can be noted among critically ill patients, the importance of a few preexisting comorbidities, which have been considered risk factors for a while. According to the USA CDC, the most critical comorbidities, and more commonly observed in fatal cases, were hypertension (48%), diabetes mellitus (31%), cardiovascular disease (13%) and age over 70 years. The average age of patients with the greatest disease severity was 52 years (40.0-65.0), the greatest severity being linked to an increase in patients' age (Wu and McGoogan 2020).

Understanding of virus-host interactions increases the chances of developing effective strategies to resolve infection. In this context, several studies have inquired the importance of variations in both *ACE2* and *TMPRSS2* expression and in individual responses to explain the wide range of symptoms and complications in COVID-19 (Lukassen *et al.* 2020; Asselta *et al.* 2020; Grifoni *et al.* 2020; Hou *et al.* 2020; Pinto *et al.* 2020). Small variations in gene expression are often related to the presence of non-pathogenic genomic variants which diverge widely between human populations (Cao *et al.* 2020). Cao and collaborators (2020) analyzed data from several large-scale genomic banks looking for variants in the *ACE2* gene associated with differences in susceptibility to SARS-CoV-2 infection in different populations. Although no direct evidence was established, the authors observed increased frequency of allelic variants of expression quantitative trait loci (eQTL) associated with increased expression of *ACE2* in East Asian populations. *ACE2* expression has also been reported to increase with age and is generally higher in men than in women (Lukassen *et al.* 2020).

In addition, increasing evidence suggests that men are more vulnerable to COVID-19 than women. Epidemiological data have shown that the majority of hospitalized patients are men in different countries (Huang *et al.* 2020; Grasselli *et al.* 2020; Guan *et al.* 2020; Richardson *et al.* 2020) and mortality also seems higher for this particular group (Jin *et al.* 2020; Onder *et al.* 2020). Many different factors have been proposed to explain the mechanisms of these discrepancies, and because behavioral differences also may play a role, the answer might not be so simple. Despite that, a "not so complex" mechanism about androgens and their part in the modulation of *TMPRSS2* expression has gathered strength. As mentioned, TMPRSS2 is a protease that cleaves the viral S protein, enabling viral and host membrane fusion. Interestingly, this protein is also known to be over expressed in more than 50% of prostate cancer patients, and it has been shown that its expression is subjected to androgen receptors activation in this organ (Vaarala *et al.* 2001). Androgen-deprivation therapies (ADTs) are a common class of treatment against prostate cancer, and recent studies showed that patients under ADT were less likely to contract COVID-19 and to be hospitalized in comparison to men that were not on ADT (Wadman 2020; Montopoli *et al.* 2020). Meanwhile, as mentioned, the degree of importance of TMPRSS2 for viral infection is not completely established, since many cell types do not express high levels of this gene/protein. Furthermore, it is still unclear whether this androgen-driven expression is also observed in the respiratory tract or in other COVID-19 relevant cell types. To investigate the molecular mechanisms of this complex sex bias, again, hiPSC models could be very helpful.

**Figure 1 -:**
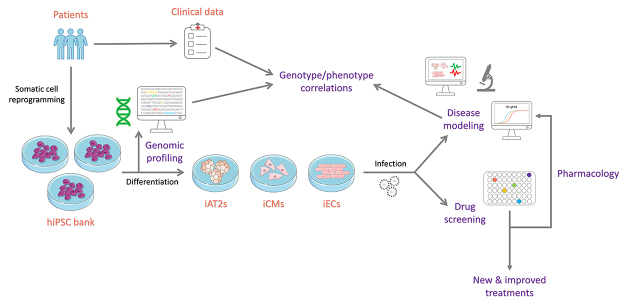
The potential uses of human induced pluripotent stem cells (hiPSCs) in COVID-19 research. Human iPS-derived cell types relevant for COVID-19 - alveolar type 2 epithelial cells (iAT2s), cardiomyocytes (iCMs) and endothelial cells (iECs), can be used for drug screening/repurposing and disease modeling. A hiPSC-bank of the Brazilian genetic admixture allows for the functional investigation of the role of host-genomics in SARS-CoV-2 infection and in COVID-19 severity.

In addition to their capability of generating several different human cell types for the study of infectious diseases, hiPSCs can also be established to broadly encompass human genetic diversity (Turner *et al.* 2013), something important to consider in the drug development process. Recognizing the diversity and genetic admixture of the Brazilian population, our group established a collection of hiPSC lineages representative of the genetic variability of our population from participants in the *ELSA*-Brazil project (*Estudo Longitudinal da Saúde de Adultos* - Longitudinal Study of Adult Health) (Tofoli *et al.* 2016). Along with its genetic admixture, each hiPSC line is connected to a large database of relevant clinical data of the corresponding participant, such as the presence of common chronic diseases like hypertension and diabetes. In the current COVID-19 pandemic scenario, in addition to the importance of clinical factors such as the occurrence of comorbidities already associated with differences in the prognosis of the infection (Guan *et al.* 2020; Zhang J *et al.* 2020; Huang *et al.* 2020; Wang T *et al.* 2020; Zhou *et al.* 2020), the genomic analysis of hiPSCs lineages’ collection can also be used in the search for polymorphisms potentially associated with the greater or lesser severity of disease (Cao *et al.* 2020; Asselta *et al.* 2020), which would provide the possibility of establishing correlations between genotype and phenotype (Figure 1).

## Conclusion

Human iPSCs are a powerful tool for the development of novel therapies for COVID-19. With their capacity to generate differentiated cells relevant for the disease, hiPSCs can be used to validate anti-viral drugs identified in large scale screens, and as an *in vitro* model system to understand the biology of host-virus interaction. In the last 15 years, the Brazilian government has made substantial investments in the development of stem cell research that can now be leveraged to contribute to research in COVID-19. Finally, the unique genetic diversity of the Brazilian population represents both a challenge and an opportunity. While findings in genomic research of virus-host interaction made in populations of European ancestry may have limited value for Brazilians, those same explorations performed in our admixed population may unravel novel genetic variants, and therefore novel molecular pathways, involved in the diverse aspects of SARS-CoV-2 infection and of COVID-19. 
